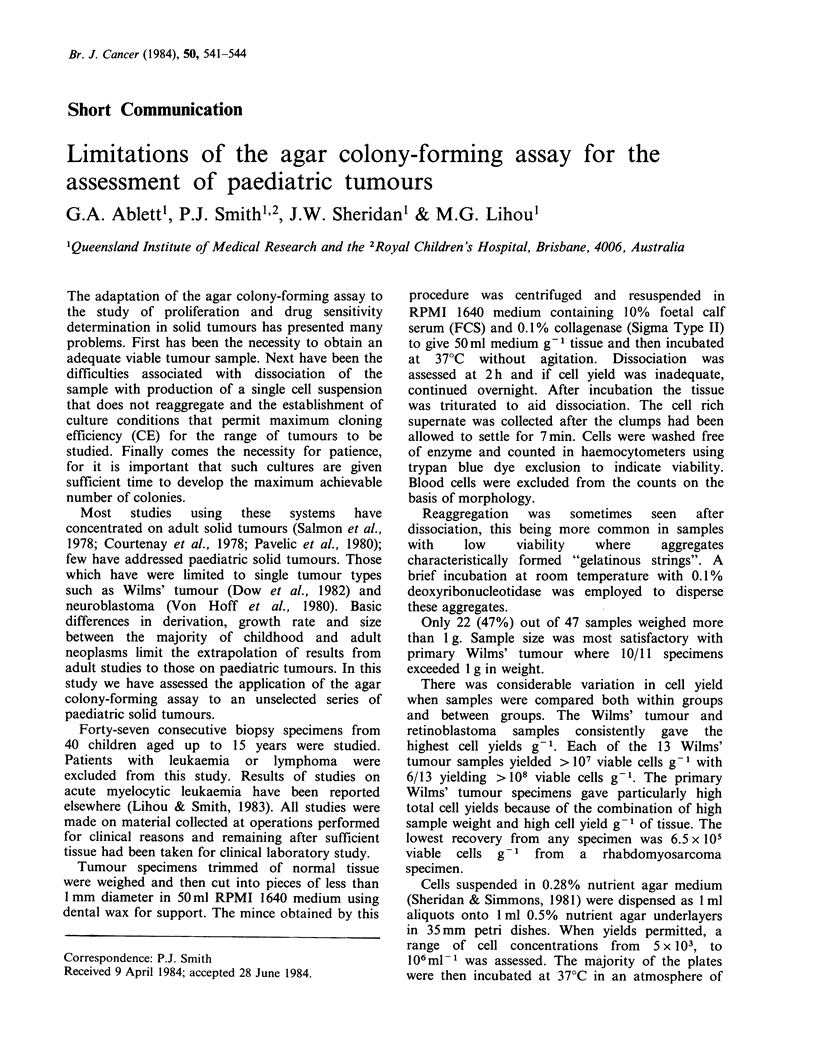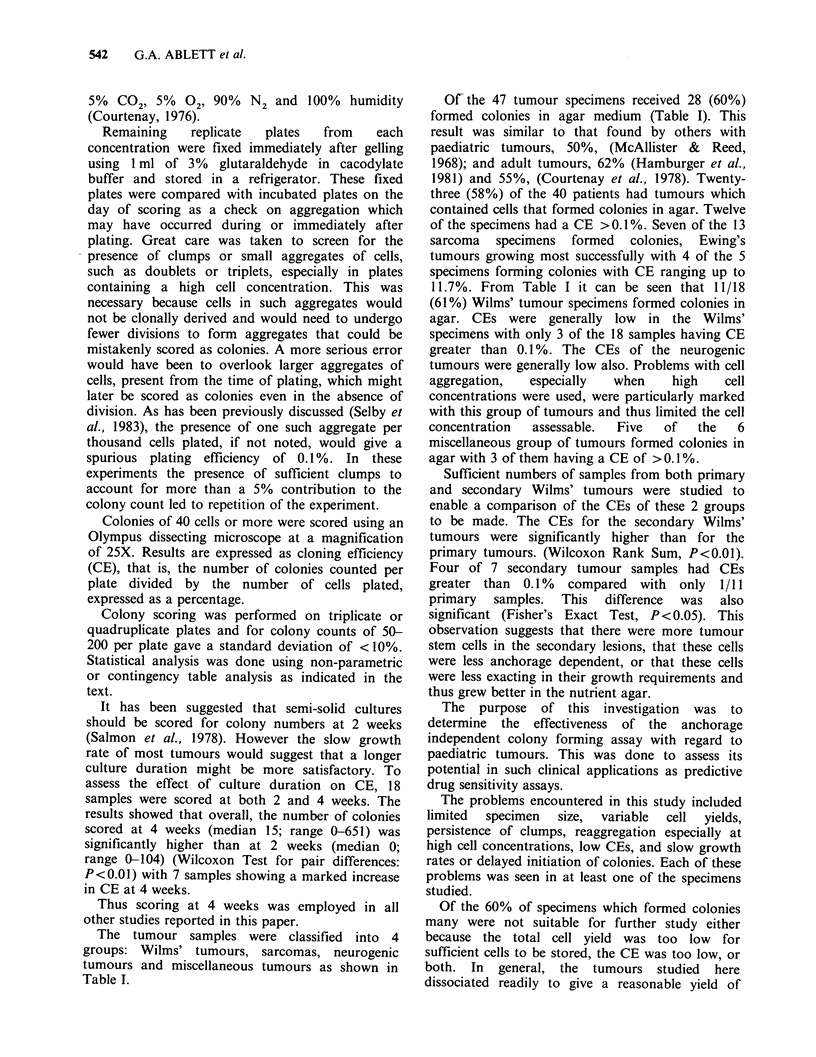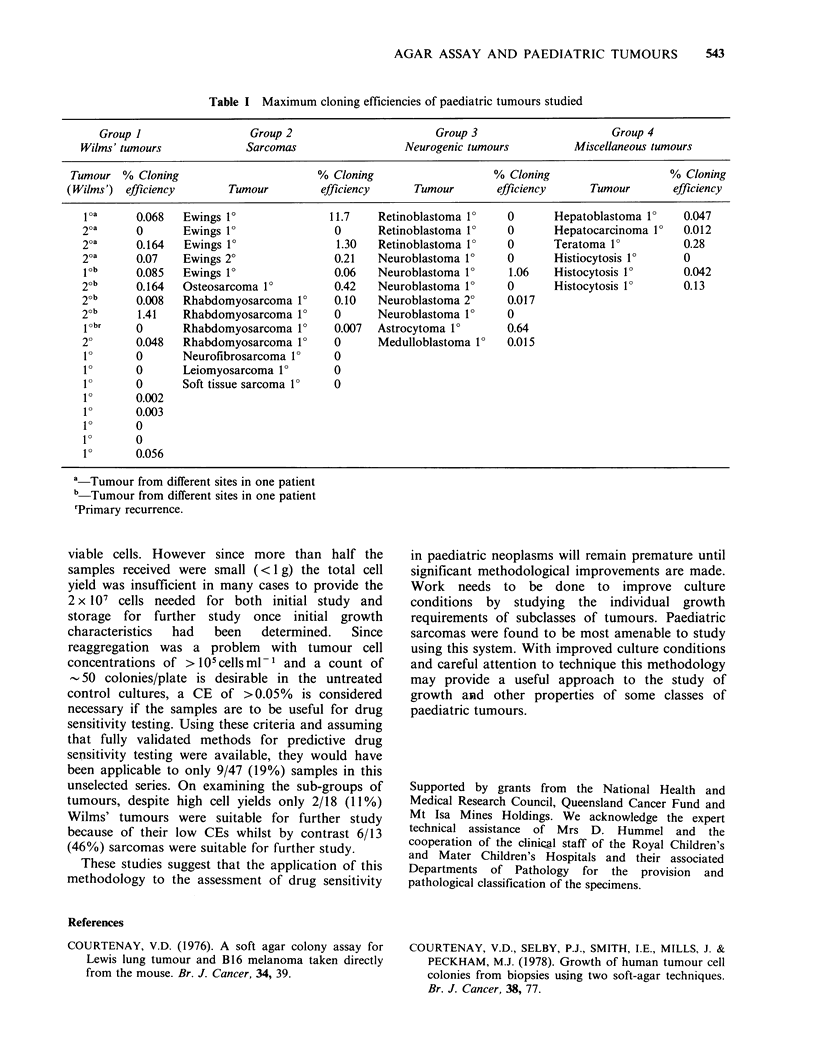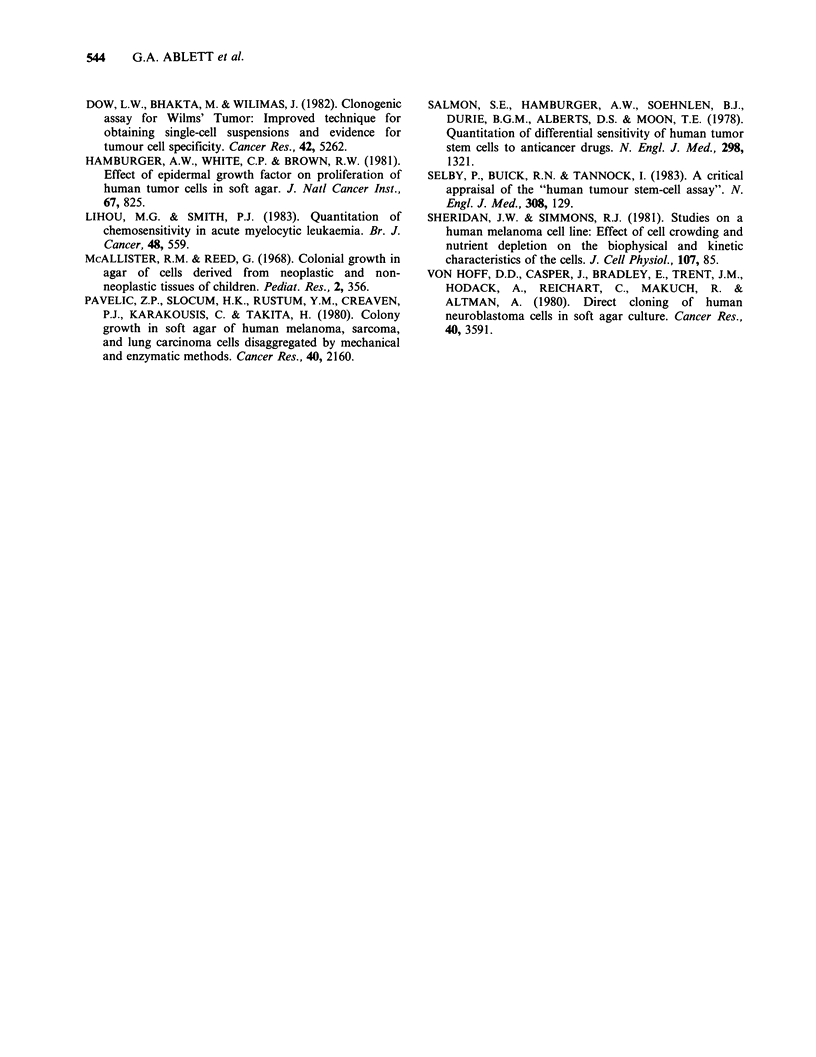# Limitations of the agar colony-forming assay for the assessment of paediatric tumours.

**DOI:** 10.1038/bjc.1984.213

**Published:** 1984-10

**Authors:** G. A. Ablett, P. J. Smith, J. W. Sheridan, M. G. Lihou


Br. J. Cancer (1984), **50**, 541–544

**Short Communication**

### Limitations of the agar colony-forming assay for the assessment of paediatric tumours

G.A. Ablett[1], P.J. Smith[1,2], J.W. Sheridan[1] & M.G. Lihou[1]

[1]*Queensland Institute of Medical Research and the* [2]*Royal Children's Hospital, Brisbane, 4006, Australia*

The adaptation of the agar colony-forming assay to the study of proliferation and drug sensitivity determination in solid tumours has presented many problems. First has been the necessity to obtain an adequate viable tumour sample. Next have been the difficulties associated with dissociation of the sample with production of a single cell suspension that does not reaggregate and the establishment of culture conditions that permit maximum cloning efficiency (CE) for the range of tumours to be studied. Finally comes the necessity for patience, for it is important that such cultures are given sufficient time to develop the maximum achievable number of colonies.

Most studies using these systems have concentrated on adult solid tumours (Salmon *et al.*, 1978; Courtenay *et al.*, 1978; Pavelic *et al.*, 1980); few have addressed paediatric solid tumours. Those which have were limited to single tumour types such as Wilms' tumour (Dow *et al.*, 1982) and neuroblastoma (Von Hoff *et al.*, 1980). Basic differences in derivation, growth rate and size between the majority of childhood and adult neoplasms limit the extrapolation of results from adult studies to those on paediatric tumours. In this study we have assessed the application of the agar colony-forming assay to an unselected series of paediatric solid tumours.

Forty-seven consecutive biopsy specimens from 40 children aged up to 15 years were studied. Patients with leukaemia or lymphoma were excluded from this study. Results of studies on acute myelocytic leukaemia have been reported elsewhere (Lihou & Smith, 1983). All studies were made on material collected at operations performed for clinical reasons and remaining after sufficient tissue had been taken for clinical laboratory study.

Tumour specimens trimmed of normal tissue were weighed and then cut into pieces of less than 1 mm diameter in 50 ml RPMI 1640 medium using dental wax for support. The mince obtained by this procedure was centrifuged and resuspended in RPMI 1640 medium containing 10% foetal calf serum (FCS) and 0.1% collagenase (Sigma Type II) to give 50 ml medium $g^{-1}$ tissue and then incubated at 37°C without agitation. Dissociation was assessed at 2 h and if cell yield was inadequate, continued overnight. After incubation the tissue was triturated to aid dissociation. The cell rich supernate was collected after the clumps had been allowed to settle for 7 min. Cells were washed free of enzyme and counted in haemocytometers using trypan blue dye exclusion to indicate viability. Blood cells were excluded from the counts on the basis of morphology.

Reaggregation was sometimes seen after dissociation, this being more common in samples with low viability where aggregates characteristically formed "gelatinous strings". A brief incubation at room temperature with 0.1% deoxyribonucleotidase was employed to disperse these aggregates.

Only 22 (47%) out of 47 samples weighed more than 1 g. Sample size was most satisfactory with primary Wilms' tumour where 10/11 specimens exceeded 1 g in weight.

There was considerable variation in cell yield when samples were compared both within groups and between groups. The Wilms' tumour and retinoblastoma samples consistently gave the highest cell yields $g^{-1}$. Each of the 13 Wilms' tumour samples yielded $>10^7$ viable cells $g^{-1}$ with 6/13 yielding $>10^8$ viable cells $g^{-1}$. The primary Wilms' tumour specimens gave particularly high total cell yields because of the combination of high sample weight and high cell yield $g^{-1}$ of tissue. The lowest recovery from any specimen was $6.5 \times 10^5$ viable cells $g^{-1}$ from a rhabdomyosarcoma specimen.

Cells suspended in 0.28% nutrient agar medium (Sheridan & Simmons, 1981) were dispensed as 1 ml aliquots onto 1 ml 0.5% nutrient agar underlayers in 35 mm petri dishes. When yields permitted, a range of cell concentrations from $5 \times 10^3$, to $10^6$ $ml^{-1}$ was assessed. The majority of the plates were then incubated at 37°C in an atmosphere of

Correspondence: P.J. Smith
Received 9 April 1984; accepted 28 June 1984.

5% $CO_2$, 5% $O_2$, 90% $N_2$ and 100% humidity (Courtenay, 1976).

Remaining replicate plates from each concentration were fixed immediately after gelling using 1 ml of 3% glutaraldehyde in cacodylate buffer and stored in a refrigerator. These fixed plates were compared with incubated plates on the day of scoring as a check on aggregation which may have occurred during or immediately after plating. Great care was taken to screen for the presence of clumps or small aggregates of cells, such as doublets or triplets, especially in plates containing a high cell concentration. This was necessary because cells in such aggregates would not be clonally derived and would need to undergo fewer divisions to form aggregates that could be mistakenly scored as colonies. A more serious error would have been to overlook larger aggregates of cells, present from the time of plating, which might later be scored as colonies even in the absence of division. As has been previously discussed (Selby et al., 1983), the presence of one such aggregate per thousand cells plated, if not noted, would give a spurious plating efficiency of 0.1%. In these experiments the presence of sufficient clumps to account for more than a 5% contribution to the colony count led to repetition of the experiment.

Colonies of 40 cells or more were scored using an Olympus dissecting microscope at a magnification of 25X. Results are expressed as cloning efficiency (CE), that is, the number of colonies counted per plate divided by the number of cells plated, expressed as a percentage.

Colony scoring was performed on triplicate or quadruplicate plates and for colony counts of 50–200 per plate gave a standard deviation of <10%. Statistical analysis was done using non-parametric or contingency table analysis as indicated in the text.

It has been suggested that semi-solid cultures should be scored for colony numbers at 2 weeks (Salmon et al., 1978). However the slow growth rate of most tumours would suggest that a longer culture duration might be more satisfactory. To assess the effect of culture duration on CE, 18 samples were scored at both 2 and 4 weeks. The results showed that overall, the number of colonies scored at 4 weeks (median 15; range 0–651) was significantly higher than at 2 weeks (median 0; range 0–104) (Wilcoxon Test for pair differences: $P<0.01$) with 7 samples showing a marked increase in CE at 4 weeks.

Thus scoring at 4 weeks was employed in all other studies reported in this paper.

The tumour samples were classified into 4 groups: Wilms' tumours, sarcomas, neurogenic tumours and miscellaneous tumours as shown in Table I.

Of the 47 tumour specimens received 28 (60%) formed colonies in agar medium (Table I). This result was similar to that found by others with paediatric tumours, 50%, (McAllister & Reed, 1968); and adult tumours, 62% (Hamburger et al., 1981) and 55%, (Courtenay et al., 1978). Twenty-three (58%) of the 40 patients had tumours which contained cells that formed colonies in agar. Twelve of the specimens had a CE >0.1%. Seven of the 13 sarcoma specimens formed colonies, Ewing's tumours growing most successfully with 4 of the 5 specimens forming colonies with CE ranging up to 11.7%. From Table I it can be seen that 11/18 (61%) Wilms' tumour specimens formed colonies in agar. CEs were generally low in the Wilms' specimens with only 3 of the 18 samples having CE greater than 0.1%. The CEs of the neurogenic tumours were generally low also. Problems with cell aggregation, especially when high cell concentrations were used, were particularly marked with this group of tumours and thus limited the cell concentration assessable. Five of the 6 miscellaneous group of tumours formed colonies in agar with 3 of them having a CE of >0.1%.

Sufficient numbers of samples from both primary and secondary Wilms' tumours were studied to enable a comparison of the CEs of these 2 groups to be made. The CEs for the secondary Wilms' tumours were significantly higher than for the primary tumours. (Wilcoxon Rank Sum, $P<0.01$). Four of 7 secondary tumour samples had CEs greater than 0.1% compared with only 1/11 primary samples. This difference was also significant (Fisher's Exact Test, $P<0.05$). This observation suggests that there were more tumour stem cells in the secondary lesions, that these cells were less anchorage dependent, or that these cells were less exacting in their growth requirements and thus grew better in the nutrient agar.

The purpose of this investigation was to determine the effectiveness of the anchorage independent colony forming assay with regard to paediatric tumours. This was done to assess its potential in such clinical applications as predictive drug sensitivity assays.

The problems encountered in this study included limited specimen size, variable cell yields, persistence of clumps, reaggregation especially at high cell concentrations, low CEs, and slow growth rates or delayed initiation of colonies. Each of these problems was seen in at least one of the specimens studied.

Of the 60% of specimens which formed colonies many were not suitable for further study either because the total cell yield was too low for sufficient cells to be stored, the CE was too low, or both. In general, the tumours studied here dissociated readily to give a reasonable yield of

**Table I** Maximum cloning efficiencies of paediatric tumours studied

| *Group 1* *Wilms' tumours* | | *Group 2* *Sarcomas* | | *Group 3* *Neurogenic tumours* | | *Group 4* *Miscellaneous tumours* | |
|---|---|---|---|---|---|---|---|
| *Tumour (Wilms')* | *% Cloning efficiency* | *Tumour* | *% Cloning efficiency* | *Tumour* | *% Cloning efficiency* | *Tumour* | *% Cloning efficiency* |
| $1^{\circ a}$ | 0.068 | Ewings 1° | 11.7 | Retinoblastoma 1° | 0 | Hepatoblastoma 1° | 0.047 |
| $2^{\circ a}$ | 0 | Ewings 1° | 0 | Retinoblastoma 1° | 0 | Hepatocarcinoma 1° | 0.012 |
| $2^{\circ a}$ | 0.164 | Ewings 1° | 1.30 | Retinoblastoma 1° | 0 | Teratoma 1° | 0.28 |
| $2^{\circ a}$ | 0.07 | Ewings 2° | 0.21 | Neuroblastoma 1° | 0 | Histiocytosis 1° | 0 |
| $1^{\circ b}$ | 0.085 | Ewings 1° | 0.06 | Neuroblastoma 1° | 1.06 | Histocytosis 1° | 0.042 |
| $2^{\circ b}$ | 0.164 | Osteosarcoma 1° | 0.42 | Neuroblastoma 1° | 0 | Histocytosis 1° | 0.13 |
| $2^{\circ b}$ | 0.008 | Rhabdomyosarcoma 1° | 0.10 | Neuroblastoma 2° | 0.017 | | |
| $2^{\circ b}$ | 1.41 | Rhabdomyosarcoma 1° | 0 | Neuroblastoma 1° | 0 | | |
| $1^{\circ br}$ | 0 | Rhabdomyosarcoma 1° | 0.007 | Astrocytoma 1° | 0.64 | | |
| 2° | 0.048 | Rhabdomyosarcoma 1° | 0 | Medulloblastoma 1° | 0.015 | | |
| 1° | 0 | Neurofibrosarcoma 1° | 0 | | | | |
| 1° | 0 | Leiomyosarcoma 1° | 0 | | | | |
| 1° | 0 | Soft tissue sarcoma 1° | 0 | | | | |
| 1° | 0.002 | | | | | | |
| 1° | 0.003 | | | | | | |
| 1° | 0 | | | | | | |
| 1° | 0 | | | | | | |
| 1° | 0.056 | | | | | | |

[a]—Tumour from different sites in one patient
[b]—Tumour from different sites in one patient
[r]Primary recurrence.

viable cells. However since more than half the samples received were small ($<1$ g) the total cell yield was insufficient in many cases to provide the $2\times10^7$ cells needed for both initial study and storage for further study once initial growth characteristics had been determined. Since reaggregation was a problem with tumour cell concentrations of $>10^5$ cells ml$^{-1}$ and a count of $\sim50$ colonies/plate is desirable in the untreated control cultures, a CE of $>0.05\%$ is considered necessary if the samples are to be useful for drug sensitivity testing. Using these criteria and assuming that fully validated methods for predictive drug sensitivity testing were available, they would have been applicable to only 9/47 (19%) samples in this unselected series. On examining the sub-groups of tumours, despite high cell yields only 2/18 (11%) Wilms' tumours were suitable for further study because of their low CEs whilst by contrast 6/13 (46%) sarcomas were suitable for further study.

These studies suggest that the application of this methodology to the assessment of drug sensitivity in paediatric neoplasms will remain premature until significant methodological improvements are made. Work needs to be done to improve culture conditions by studying the individual growth requirements of subclasses of tumours. Paediatric sarcomas were found to be most amenable to study using this system. With improved culture conditions and careful attention to technique this methodology may provide a useful approach to the study of growth and other properties of some classes of paediatric tumours.

Supported by grants from the National Health and Medical Research Council, Queensland Cancer Fund and Mt Isa Mines Holdings. We acknowledge the expert technical assistance of Mrs D. Hummel and the cooperation of the clinical staff of the Royal Children's and Mater Children's Hospitals and their associated Departments of Pathology for the provision and pathological classification of the specimens.

**References**

COURTENAY, V.D. (1976). A soft agar colony assay for Lewis lung tumour and B16 melanoma taken directly from the mouse. *Br. J. Cancer*, **34**, 39.

COURTENAY, V.D., SELBY, P.J., SMITH, I.E., MILLS, J. & PECKHAM, M.J. (1978). Growth of human tumour cell colonies from biopsies using two soft-agar techniques. *Br. J. Cancer*, **38**, 77.

DOW, L.W., BHAKTA, M. & WILIMAS, J. (1982). Clonogenic assay for Wilms' Tumor: Improved technique for obtaining single-cell suspensions and evidence for tumour cell specificity. *Cancer Res.*, **42,** 5262.

HAMBURGER, A.W., WHITE, C.P. & BROWN, R.W. (1981). Effect of epidermal growth factor on proliferation of human tumor cells in soft agar. *J. Natl Cancer Inst.*, **67,** 825.

LIHOU, M.G. & SMITH, P.J. (1983). Quantitation of chemosensitivity in acute myelocytic leukaemia. *Br. J. Cancer,* **48,** 559.

McALLISTER, R.M. & REED, G. (1968). Colonial growth in agar of cells derived from neoplastic and non-neoplastic tissues of children. *Pediat. Res.*, **2,** 356.

PAVELIC, Z.P., SLOCUM, H.K., RUSTUM, Y.M., CREAVEN, P.J., KARAKOUSIS, C. & TAKITA, H. (1980). Colony growth in soft agar of human melanoma, sarcoma, and lung carcinoma cells disaggregated by mechanical and enzymatic methods. *Cancer Res.*, **40,** 2160.

SALMON, S.E., HAMBURGER, A.W., SOEHNLEN, B.J., DURIE, B.G.M., ALBERTS, D.S. & MOON, T.E. (1978). Quantitation of differential sensitivity of human tumor stem cells to anticancer drugs. *N. Engl. J. Med.*, **298,** 1321.

SELBY, P., BUICK, R.N. & TANNOCK, I. (1983). A critical appraisal of the "human tumour stem-cell assay". *N. Engl. J. Med.*, **308,** 129.

SHERIDAN, J.W. & SIMMONS, R.J. (1981). Studies on a human melanoma cell line: Effect of cell crowding and nutrient depletion on the biophysical and kinetic characteristics of the cells. *J. Cell Physiol.*, **107,** 85.

VON HOFF, D.D., CASPER, J., BRADLEY, E., TRENT, J.M., HODACK, A., REICHART, C., MAKUCH, R. & ALTMAN, A. (1980). Direct cloning of human neuroblastoma cells in soft agar culture. *Cancer Res.*, **40,** 3591.